# p53 Function and Dysfunction in Human Health and Diseases

**DOI:** 10.3390/biom13030506

**Published:** 2023-03-09

**Authors:** Gabriella D’Orazi

**Affiliations:** 1 Unit of Cellular Networks, Department of Research, IRCCS Regina Elena National Cancer Institute, 00144 Rome, Italy; gdorazi@unich.it; 2 Department of Neurosciences, Images and Clinical Sciences, University G. D’Annunzio, 00131 Chieti, Italy

The p53 protein is a sequence-specific DNA-binding transcription factor that, in response to stressful stimuli, regulates gene expression related to multiple cellular functions including, but not limited to, cell cycle arrest, cell apoptosis, cell growth, DNA repair, cell metabolism, and the immune response [1]. The *TP53* gene is often mutated in cancers, mostly in the DNA-binding (DBD) domain, to avoid binding to canonical target gene promoters; however, many other mechanisms may impair the wild-type p53 function by deregulating p53 at the protein level [2]. The perturbation of p53 signaling pathways is required for the development of most cancers; however, it is now becoming clear that p53 has a much broader role in human health and diseases. Knowing the mechanisms leading to p53 deregulation may pave the way to a better understanding of tumor progression and the development of novel therapeutic strategies against cancer [3].

This Special Issue includes eight original research papers and three reviews from experts in the field, providing the reader with advances in the understanding of how the perturbation of p53 activity, via gene mutation, alteration of p53 protein regulation or interaction with oncogenic pathways, might be a key determinant for tumor progression and chemoresistance. The articles also summarize the most recently developed therapeutic strategies.

The review by Zhang et al. summarizes the current progress in targeting wild-type and mutant p53 for cancer therapy through biotherapeutic and biopharmaceutical methods starting from the analysis of p53′s structure and mutations exploited for drug development [4]. *TP53* mutations generally occur in the DNA-binding domain, with approximately 30% arising in specific hotspots, including R175, G245, R248, R249, R273, and R282. Clinical studies have demonstrated that in many tumor types, the presence of mutated p53 correlates with a worse patient prognosis as compared to the presence of wild-type p53. p53 mutations vary greatly in terms of their impact on p53 function. Some mutations cause loss of p53 function, some provide p53 with the ability to inactivate wild-type p53 that is expressed by the remaining wild-type allele (dominant-negative p53 mutations), and others cause p53 to acquire oncogenic gain-of-function (GOF) properties. These different effects make mutant p53 a challenging drug target for prognostic and therapeutic purposes [5]. For example, mutations in residues such as Arg273 and Arg282 destabilize the core DNA-binding domain by enhancing the thermodynamic and kinetic instability of mutant p53. These effects are often exploited to design ligands that selectively bind to the native state of the p53 protein to reverse the thermodynamic and kinetic denaturation consequences of these mutations. On the other hand, R175H has been described as a zinc-binding mutant, and small molecules functioning as zinc metallochaperones may stabilize mutant p53-Zn^2+^ interaction, resulting in a wild-type conformational change with restoration of wild-type p53 function in vitro and in vivo [6,7,8]. Another way to target mutant p53 is via the induction of mutant p53 degradation via the proteasome or lysosome [9,10,11]. The review by Zhang et al. summarizes the strategies for boosting wild-type p53 activity in cancer. These strategies include gene therapy, cytotoxic chemotherapy, MDM2/MDMX/MDM4 inhibitors, p53-binding compounds, and targeting of p53 post-translational modifications. Many efforts over the past two decades have been dedicated to the development of new therapeutic approaches able to reactivate mutant p53 or to restore wild-type p53 activity in order to re-establish cancer cell chemosensitivity and improve immunotherapy. Although there are no FDA-approved drugs targeting p53 (wild-type or mutant), these pioneering studies shed light on prospective cancer therapy that targets mutant and wild-type p53 [4].

In the study by Romeo et al., the authors showed that overexpression of the p53-R273H mutant protein in p53-null cancer cells promotes cancer cell survival by increasing intracellular ROS levels and pro-inflammatory/immune-suppressive cytokine release. P53-R273 overexpression activates the mTOR pro-survival pathway while reducing autophagy and mitophagy. Interestingly, p53-R273H transfection into cancer cells carrying wtp53 induces none of these effects, suggesting that wtp53 may counteract several pro-tumorigenic activities of p53-R273H; this could explain the lower aggressiveness of cancers carrying heterozygous mutp53 in comparison to those harboring homozygous mutp53 [12].

The challenges and prospects of p53-based therapies in ovarian cancer are summarized in the review by Wallis et al. [13]. Ovarian cancer is the fifth leading cause of death in women, despite accounting for only 2.5% of all female malignancies. The overall 5-year survival rate for ovarian cancer is around 47%; however, this drops to an abysmal 29% for the most common type of ovarian cancer, high-grade serous ovarian carcinoma (HGSOC), in which upwards of 96% of cases expressing mutations in p53. The review highlights a selection of the historical p53-targeted therapeutics for ovarian cancer, why they failed, and what the future could hold for a new generation of this class of therapies [13].

Faustino da Silva et al. investigated the prevalence of *TP53* p.R337H germline pathogenic variants in a cohort of 83 breast cancer (BC) patients from midwestern Brazilian, where this variant is highly prevalent [14]. All patients met the clinical criteria for hereditary breast and ovarian cancer syndrome (HBOC) and were negative for *BRCA1* and *BRCA2* mutation. The study identified 4 Li–Fraumeni syndrome patients out of 40 within the age range of 22–24 who met the Chompret criteria with germline *TP53* pathogenic variants including the founder mutation p.R337H, while the remaining 36 patients tested negative for *BRCA1*, *BRCA2*, and *TP53* pathogenic variants. This study provides useful information on TP53 Li–Fraumeni syndrome (LFS)/Li–Fraumeni-like (LFL) HBOC in midwestern Brazil.

Wild-type p53 can also be activated through MDM2-mediated protein degradation [15]. The effect of targeting MDM2/p53 interaction was evaluated in Hanningsen’s study. Upon induction by p53, the MDM2 protein binds and ubiquitinates p53, triggering its proteasomal degradation and providing negative feedback. The study considered whether MDM2 can also remove p53 from its target promoters, and whether this involves ubiquitination. The study employed the MDM2-targeted small molecule Nutlin-3a to disrupt the interaction of MDM2 and p53 in three different cancer cell lines. The results strongly suggest that MDM2-mediated ubiquitination not only triggers p53 degradation but also the removal of p53 from its bond with promoter DNA within minutes [16]. The question remains whether this activity of MDM2, i.e., its ability to dissociate p53 from DNA, can be regulated under physiological circumstances. It is tempting to speculate that many regulatory pathways acting on p53 and MDM2 for p53 ubiquitination may not only affect the degradation of p53 but also directly govern the association of p53 with DNA. In another study, the authors found a promising mechanism leading to MDM2-mediated p53 inactivation. They found that CCR4-NOT transcription complex subunit 2 (CNOT2) is overexpressed in colon cancer patients and cells correlating with MDM2 upregulation, and that CNOT2 knockout induces p53 protein stability and restores p53 apoptotic activity [17]. These findings suggest that CNOT2 plays an oncogenic role in human cancer cells through the inactivation of p53, which is worth further study.

A number of p53-MDM2 inhibitors have been tested preclinically in neuroblastomas with positive results. The novel MDM2/MDM4-p53 antagonist peptide VIP116 was tested as a monotherapy and together with ^177^Lu-DOTATATE in both commercially available cell lines and alongside the use of patient-derived xenograft (PDX) models established from neuroblastoma patients [18]. This heterogenous group of pediatric tumors includes a subset of high-risk patients in need of more therapeutic alternatives. The study demonstrates that p53 stabilization with VIP116, as well as targeted radionuclide therapy with ^177^Lu-DOTATATE, are feasible treatment options for neuroblastomas. Moreover, the combination of VIP116 and ^177^Lu-DOTATATE is particularly promising, due to its synergistic effects [18].

Social environment can impact tumor progression in humans. Despite this epidemiological evidence, few studies have directly investigated this issue, and the underlying biological mechanisms remain largely unknown. Middei et al., using a combination of enrichment conditions and DNA-damage-mediated tumor induction, reported that exposing mice pups to early physical and social enrichment delays tumor progression in adulthood. In agreement with these results on a molecular level, the authors observed increased p53 activity in healthy tissue and decreased levels of Mdm2, the main inhibitor of p53, suggesting that early social enrichment can antagonize DNA-damage-induced tumorigenesis in mice with the possible involvement of p53 activity [19]. These findings are in line with a set of studies demonstrating that exposure to protective factors can drive persistent changes in molecular pathways, which are sufficient to trigger anti-tumoral responses upon carcinogenic insult [20].

Wild-type p53 oncosuppressor activity may be impaired by antioxidant pathways driven by the NRF2 transcription factor [21]. Garufi et al. investigated the response of stable NRF2 knockout in colon cancer cells to chemotherapeutic drugs with regard to p53 oncosuppressor activity. The results highlight the key role of NRF2 in neutralizing the cytotoxic effects of chemotherapeutic drugs in correlation with reduced DNA damage and p53 activity. They also suggest that NRF2 inhibition could be a useful strategy for improving the effect of chemotherapy in combination with zinc supplementation to restore p53 apoptotic activity [22].

Lately, it has become evident that mutant p53 can engage in interplay with the cellular stress pathway, promoting cancer progression [23]. In this regard, the study by Xiao et al. provides a comprehensive overview of molecular interactions between p53 and the Wnt pathway, including complex feedback loops and reciprocal transactivation. They describe the mutational landscape of genes associated with p53 and Wnt signaling and summarize the functional consequences of this interplay in cancer progression, including invasiveness, metastasis, and drug resistance, and discuss potential strategies to pharmacologically target p53-Wnt interaction [24]. As therapeutic strategies directed against p53-Wnt pathways have been largely unsuccessful in the past, understanding the tissue and mutational contexts of the interaction would help in developing more tailored and efficient treatment strategies against cancer.

p53′s tumor-suppressive activity has been found to limit tumor cell proliferation not only within cells but also in the extracellular space. Thus, the loss of p53 has a profound impact on the secretome composition of cancer cells and marks the transition to invasiveness. The wild-type p53 protein, which is frequently mutated in pancreatic ductal adenocarcinoma (PDAC), prevents tumorigenesis by regulating a plethora of signaling pathways. The study of the cancer secretome is gaining even more importance in cancers such as PDAC, whose lack of recognizable symptoms and early detection assays makes it highly lethal. Butera et al. investigated the tumor-suppressive role of wild-type p53 on the cancer cell secretome of PDAC, showing the anti-proliferative, apoptotic, and chemosensitivity effects of a conditioned medium driven by wild-type p53. Using high-resolution SWATH-MS technology, they characterized the secretomes of p53-deficient and p53-expressing PDAC cells. They found a great number of secreted proteins that have known roles in cancer-related processes, 30 of which showed enhanced secretion and 17 reduced secretion in response to p53 silencing. These results advance the understanding of the link between wt-p53 and cancer microenvironment, and they also may be useful in detecting a secreted signature specifically driven by wild-type p53 in PDAC [25].

We hope that the readers will enjoy reading this Special Issue of *Biomolecules* and that the findings presented here will help advance the understanding of the impact that p53 deregulation has on cancer and encourage new research aimed at finding novel anti-cancer strategies able to reactivate wild-type p53 oncosuppressor function and/or to block mutant p53 function, in order to improve cancer treatment and prognosis.

## References

[1] Kastenhuber E.R., Lowe S.W. (2017). Putting p53 in Context. Cell.

[2] Muller P.A., Vousden K.H. (2014). Mutant p53 in cancer: New functions and therapeutic opportunities. Cancer Cell.

[3] Sabapathy K., Lane D. (2018). Therapeutic targeting of p53: All mutants are equal, but some mutants are more equal than others. Nat. Rev. Clin. Oncol..

[4] Zhang S., Carlsen L., Hernandez Borrero L., Seyhan A.A., Tian X., El-Deiry W.S. (2022). Advanced strategies for therapeutic targeting of wild-type and mutant p53 in cancer. Biomolecules.

[5] Schulz-Heddergott R., Moll U.M. (2018). Gain-of-function (GOF) mutant p53 are actionable therapeutic target. Cancers.

[6] Puca R., Nardinocchi L., Porru M., Simon A.S., Rechavi G., Leonetti C., Givol D., D’Orazi G. (2011). Restoring p53 active conformation by zinc increases the response of mutant p53 tumor cells to anticancer drugs. Cell Cycle.

[7] Yu X., Vazquez A., Levine A.J., Carpizo D.R. (2012). Allele-Specific p53 Mutant Reactivation. Cancer Cell.

[8] Cirone M., Garufi A., Di Renzo L., Franato M., Faggioni A., D’Orazi G. (2013). Zinc supplementation is required for the cytotoxic and immunogenic effects of chemotherapy in chemoresistant p53-functionally deficient cells. Oncoimmunology.

[9] Vakifahmetoglu-Norberg H., Kim M., Xia H.-G., Iwanicki M.P., Ofengeim D., Coloff J., Pan L., Ince T.A., Kroemer G., Brugge J.S. (2013). Chaperone-mediated autophagy degrades mutant p53. Genes Dev..

[10] Rodriguez O.C., Choudhury S., Kolukula V., Vietsch E.E., Catania J., Preet A., Reynoso K., Bargnetti J., Wellstein A., Albanese C. (2012). Dietary downregulation of mutant p53 levels via glucose restriction: Mechanisms and implications for tumor therapy. Cell Cycle.

[11] Garufi A., Pucci D., D’Orazi V., Cirone M., Bossi G., Avantaggiti M.L., D’Orazi G. (2014). Degradation of mutant p53H175 protein by Zn(II) through autophagy. Cell Death Dis..

[12] Romeo M.A., Gilardini Montani M.S., Benedetti R., Arena A., D’Orazi G., Cirone M. (2021). p53-R273H sustains ROS, pro-inflammatory cytokines release and mTOR activation while reducing autophagy, mitophagy and UCP2 expression, effects prevented by wtp53. Biomolecules.

[13] Wallis B., Bowman K.R., Lu P., Lim C.S. (2023). The challenges and prospects of p53-based therapies in ovarian cancer. Biomolecules.

[14] Da Silva P.F.F., Mota Goveia R., Bomfim Teixeira T., Faulin Gamba B., de Lima A.P., Rogatto S.R., de Paula Silvera-Lacerda E. (2022). TP53 pathogenic variants in early-onset breast cancer patients fulfilling hereditary breast and ovarian cancers and Li-Fraumeni-like syndromes. Biomolecules.

[15] Haupt Y., Maya R., Kazaz A., Oren M. (1997). Mdm2 promotes the rapid degradation of p53. Nature.

[16] Henningsen K.M., Manzini V., Magerhans A., Gerber S., Dobbelstein M. (2021). MDM2-driven ubiquitination rapidly removes p53 from its cognate promoters. Biomolecules..

[17] Jung J.H., Lee D., Ko H.M., Jang H.-J. (2021). Inhibition of CNOT2 induces apoptosis via MID1IP1 in colorectal cancer cells by activating p53. Biomolecules.

[18] Lundsten S., Berglund H., Jha P., Krona C., Hariri M., Nelander S., Lane D.P., Nestor M. (2021). p53-mediated radiosensitization of ^177^Lu-DOTATATE in neuroblastoma tumor spheroids. Biomolecules.

[19] Middei S., Giorgini L., Vacca V., Storri F., Putti S., Strimpakos G., Raspa M., Scavizzi F., Moretti F., D’Amato F.R. (2022). Early social enrichment modulates tumor progression and p53 expression in adult mice. Biomolecules.

[20] Sivaraman L., Conneely O.M., Medina D., O’Malley B.W. (2001). p53 is a potential mediator of pregnancy and hormone-induced resistance to mammary carcinogenesis. Proc. Natl. Acad. Sci. USA.

[21] Cirone M., D’Orazi G. (2023). NRF2 in cancer: Croos-talk with oncogenic pathways and involvement in gamma herpesviruses-driven carcinogenesis. Int. J. Mol. Sci..

[22] Garufi A., Pistritto G., D’Orazi V., Cirone M., D’Orazi G. (2022). The impact of NRF2 inhibition on drug-induced colon cancer cell death and p53 activity: A pilot study. Biomolecules.

[23] D’Orazi G., Cirone M. (2019). Mutant p53 and cellular stress pathways: A criminal alliance that promotes cancer progression. Cancers.

[24] Xiao Q., Werner J., Venkatachalam N., Boonekamp K.E., Ebert M.P., Zhan T. (2022). Cross-talk between p53 and Wnt signaling in cancer. Biomolecules.

[25] Butera G., Manfredi M., Fiore A., Brandi J., Pacchiana R., De Giorgis V., Barberis E., Vanella V., Galasso M., Scupoli M.T. (2022). Tumor suppressor role of wild-type p53-dependent secretome and its proteomic identification in PDAC. Biomolecules.

